# The Phylogeographical Pattern and Conservation of the Chinese Cobra (*Naja atra*) across Its Range Based on Mitochondrial Control Region Sequences

**DOI:** 10.1371/journal.pone.0106944

**Published:** 2014-09-03

**Authors:** Long-Hui Lin, Lei Hua, Yan-Fu Qu, Jian-Fang Gao, Xiang Ji

**Affiliations:** 1 Hangzhou Key Laboratory for Animal Adaptation and Evolution, School of Life Sciences, Hangzhou Normal University, Hangzhou, Zhejiang, China; 2 Jiangsu Key Laboratory for Biodiversity and Biotechnology, College of Life Sciences, Nanjing Normal University, Nanjing, Jiangsu, China; Institute of Biochemistry and Biology, Germany

## Abstract

The vulnerable Chinese cobra (*Naja atra*) ranges from southeastern China south of the Yangtze River to northern Vietnam and Laos. Large mountain ranges and water bodies may influence the pattern of genetic diversity of this species. We sequenced the mitochondrial DNA control region (1029 bp) using 285 individuals collected from 23 localities across the species' range and obtained 18 sequences unique to Taiwan from GenBank for phylogenetic and population analysis. Two distinct clades were identified, one including haplotypes from the two westernmost localities (Hekou and Miyi) and the other including haplotypes from all sampling sites except Miyi. A strong population structure was found (Φst = 0.76, *P*<0.0001) with high haplotype diversity (*h* = 1.00) and low nucleotide diversity (*π* = 0.0049). The Luoxiao and Nanling Mountains act as historical geographical barriers limiting gene exchange. In the haplotype network there were two “star” clusters. Haplotypes from populations east of the Luoxiao Mountains were represented within one cluster and haplotypes from populations west of the mountain range within the other, with haplotypes from populations south of the Nanling Mountains in between. Lineage sorting between mainland and island populations is incomplete. It remains unknown as to how much adaptive differentiation there is between population groups or within each group. We caution against long-distance transfers within any group, especially when environmental differences are apparent.

## Introduction

Cobras of the genus *Naja* are a clade of elapid snakes that occur in Africa and Asia. They have attracted worldwide attention for their unique hood-spreading behavior and deadly toxicity. The genus currently contains 28 oviparous species in four subgenera: *Afronaja*, *Boulengerina*, *Naja* and *Uraeus* [1], of which all but *Naja* are primarily African. The 11 Asian cobras in the subgenus *Naja* are thought to comprise a lineage that spreads from west to east, from Africa through India [2]–[4]. Two *Naja* cobras can be found in China, the Chinese cobra *N. atra* and the monocled cobra *N. kaouthia* [5]. *Naja atra* occurs in the southeastern provinces (including Taiwan) of China south of 30°17′N north latitude, northern Vietnam and Laos; *N. kaouthia* ranges from southwestern China to south-southeastern Asia [1], [6]–[8].

In China, *N. atra* is much more widely distributed and commonly found than *N. kaouthia*, and is among the snake species that have long been used by local people as traditional food and medicine. The long-term unsustainable exploitation of *N. atra* has made it one of the most vulnerable snake species [5]. The immense and increasingly rampant illegal trade of *N. atra* has not only exacerbated the decline of wild populations, but also disrupted their lineage relationships due to escapes and uncontrolled releases of wild-caught individuals. As a “top-of-the-food-chain” predator, *N. atra* is important to its ecosystem because it can keep the populations of its various prey species (e.g., rodents and toads) under control [5]. Conservation measures should be taken immediately to protect this ecologically important cobra [9].

Understanding population structure and genetic diversity is valuable for wildlife conservation because of its importance in determining the integrity and viability of natural populations. Previous studies on *N. atra* have shown that population genetic structure is correlated with geography. Using mitochondrial DNA markers (cyt *b* and ND2) to examine relationships among five mainland populations, Lin et al. (2008) found that three eastern populations (two in Zhoushan and Lishui of Zhejiang Province, and one in Huangshan of Anhui Province) cluster together, while two southern populations in Baise and Quanzhou of Guangxi Province form another clade [10]. Sequencing the mitochondrial control region from 127 individuals from four populations in northern, central, southern and western parts of Taiwan, HC Lin et al. (2008) found that overall nucleotide diversity is low and gene flow between populations is not obvious [11]. Therefore, they suggested the division of *N. atra* in Taiwan into four independent evolutionary significant units. Combining mitochondrial cyt *b* gene and microsatellite markers, Lin et al. (2012) found that: (1) mitochondrial DNA data reveal two main (Vietnam + southern China + southwestern China; eastern + southeastern China) and one minor (comprising only two individuals from the westernmost site) clades; (2) microsatellite data divide the eastern + southeastern China clade further into two genetic clusters; and (3) the Luoxiao and Nanling Mountains are inferred to be the major barriers causing lineage sorting [9]. Accordingly, Lin et al. (2012) suggested the division of *N. atra* in mainland China into three independent management units [9]. Existing studies on *N. atra* conducted independently in mainland China and Taiwan have been limited by incomplete sampling, not allowing the development of a generally applicable conservation plan for the species. Thus, further work through more intensive and large-scale sampling within the species' entire range by using the same genetic markers is needed to reveal its overall genetic diversity and population genetic structure. Our aim is to find different lineages across this vulnerable snake's range and take them into account for conservation plans.

## Results

### Genetic diversity and genealogy

The 1029 bp mitochondrial control region sequences (GenBank Accession Numbers: DQ224315-316, DQ224318-323, DQ224326-337, GU563498-526, HQ881480-483) were obtained from 390 individuals (of which 105 were from Taiwan [11]) after alignment. A total of 40 variable nucleotide sites (of which 18 were parsimony informative), comprising 32 transitions (ts) and 9 transversions (tv) (both ts and tv at the 853^th^ site), defined 52 haplotypes. Sixteen haplotypes were unique to a single population. The number of haplotypes within each locality ranged from 1 to 12 (Table 1). Haplotype diversity (*h*) ranged from 0 (the Huangshan, Lishui, Jianyang, Ganzhou, Miyi, Zhijiang and Songtao populations) to 0.920 (the southern Taiwan population), and nucleotide diversity (*π*) from 0 (the Huangshan, Lishui, Jianyang, Ganzhou, Miyi, Zhijiang and Songtao populations) to 0.0057 (the Hekou population) (Table 1). For the whole sample, *h* was 1.000 and *π* was 0.0049, indicating high haplotype diversity but relatively low nucleotide diversity.

**Table 1:** Samples sites, number of haplotypes (*N*), haplotype diversity (*h*), nucleotide diversity (*π*), Fu's *Fs*, *τ*, sum of squared deviation (SSD), and Harpending's raggedness index (HRI) for *Naja. atra*.

Code	Sample site	Genetic diversity			Neutrality	Mismatch distribution		
		*N*	*h*	*π* (%)	Fu's *Fs*	SSD	HRI	*τ*
TE	Eastern Taiwan^E^	5	0.649	0.077	−1.613^NS^	–	–	
TN	Northern Taiwan^E^	6	0.556	0.068	−2.865*	–	–	
TW	Western Taiwan^E^	8	0.800	0.149	−2.176^NS^	0.006*	0.054***	
TS	Southern Taiwan^E^	12	0.920	0.227	−5.633**	–	–	
ZS	Zhoushan islands^E^	2	0.514	0.100	2.710^NS^	0.195***	0.765***	
HS	Huangshan^E^	1	0	0	–	–	–	
LI	Lishui^E^	1	0	0	–	–	–	
JY	Jianyang^E^	1	0	0	–	–	–	
YA	Yong'an^E^	2	0.250	0.024	−0.182^NS^	0.001^NS^	0.313^NS^	
YD	Yongding^E^	2	0.327	0.032	0.356^NS^	0.003^NS^	0.226^NS^	
JA	Ji'an^E^	4	0.821	0.146	−0.422^NS^	–	–	
GZ	Ganzhou^E^	1	0	0	–	–	–	
WY	Wengyuan^S^	5	0.818	0.214	0.005^NS^	0.050^NS^	0.164^NS^	
ZQ	Zhaoqing^S^	7	0.886	0.283	−0.779^NS^	0.018*	0.075***	
HN	Hainan island^S^	3	0.530	0.159	2.884^NS^	0.233***	0.639^NS^	
VN	Vietnam^S^	5	0.782	0.253	0.588^NS^	0.178***	0.515***	
BS	Baise^S^	3	0.714	0.167	1.243^NS^	0.060^NS^	0.204^NS^	
HK	Hekou^W^	4	0.725	0.572	5.544^NS^	0.099***	0.260*	
MY	Miyi^W^	1	0	0	–	–	–	
GL	Guanling^W^	2	0.133	0.039	0.834^NS^	0.026***	0.787^NS^	
LS	Leishan^W^	3	0.676	0.098	0.948^NS^	0.013^NS^	0.112^NS^	
QZ	Quanzhou^W^	2	0.525	0.051	1.333^NS^	–	–	
CZ	Chenzhou^W^	2	0.429	0.042	0.536^NS^	–	–	
YZ	Yongzhou^W^	3	0.641	0.080	0.375^NS^	–	–	
ZJ	Zhijiang^W^	1	0	0	–	–	–	
ST	Songtao^W^	1	0	0	–	–	–	
JS	Jinshi^W^	2	0.400	0.039	0.872^NS^	–	–	
E: Eastern populations		26	0.804	0.175	−16.854***	0.001^ NS^	0.028^ NS^	1.824
S: Southern populations		19	0.903	0.306	−5.974*	0.020 *	0.060*	
W: Western populations		13	0.885	0.498	2.366^ NS^	0.010^ NS^	0.016^ NS^	

Superscripts denote population sources. *0.05 ≧ *P* ≧ 0.01; **0.01>*P* ≧ 0.001; ***P<0.001; NS, not significant

**Figure 1:**
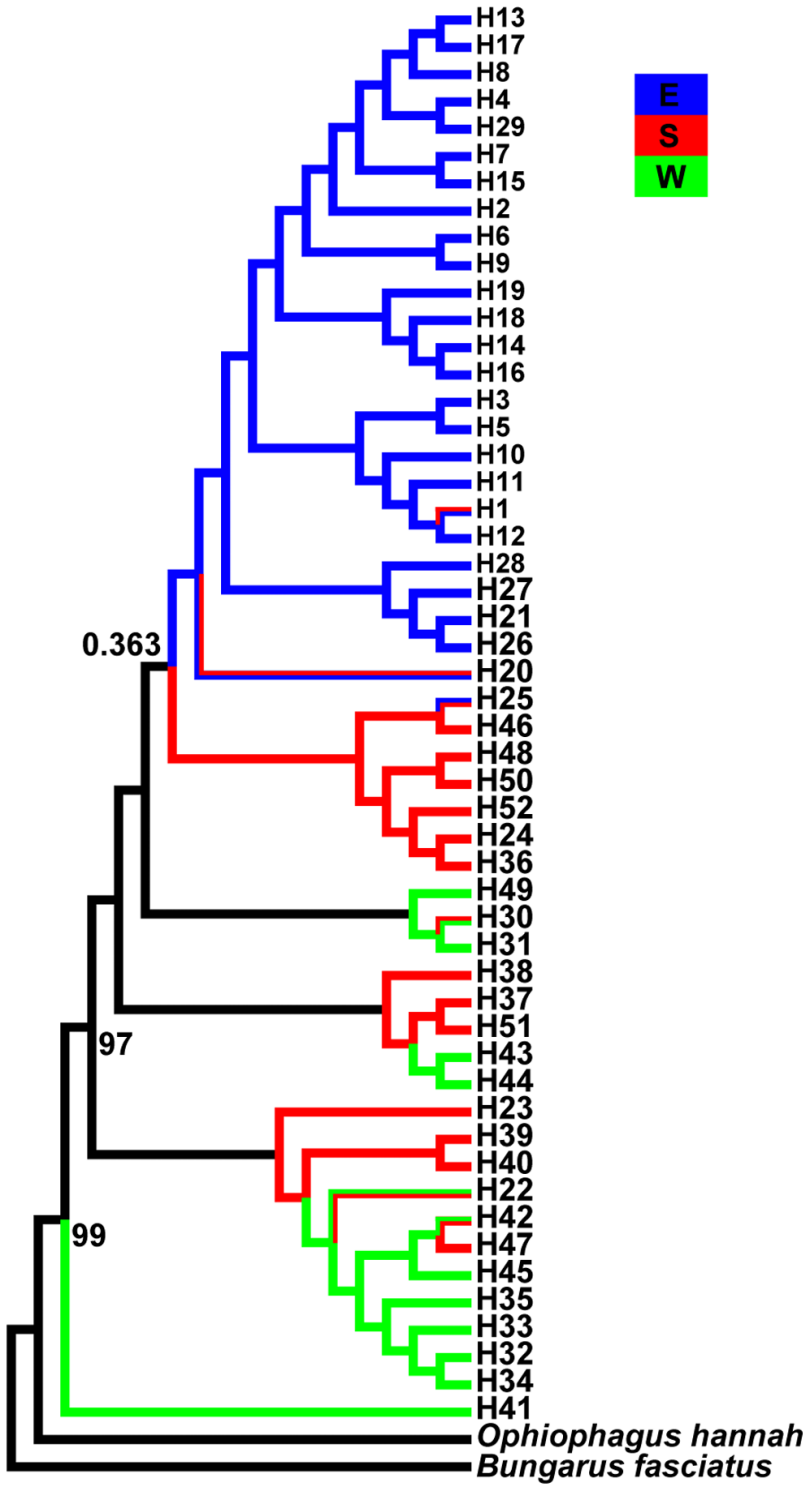
Maximum-likelihood tree for all 52 haplotypes of *Naja atra*, with *Ophiophagus hannah* and *Bungarus fasciatus* as the outgroup taxa. Labels are haplotype identification numbers. Values on the right side of the nodes indicate support for each node based on maximum likelihood. Bootstrap values below 60% are not shown. The value of 0.363 mya above the branch indicates the divergence time between the eastern population groups and the other two population groups.

**Figure 2:**
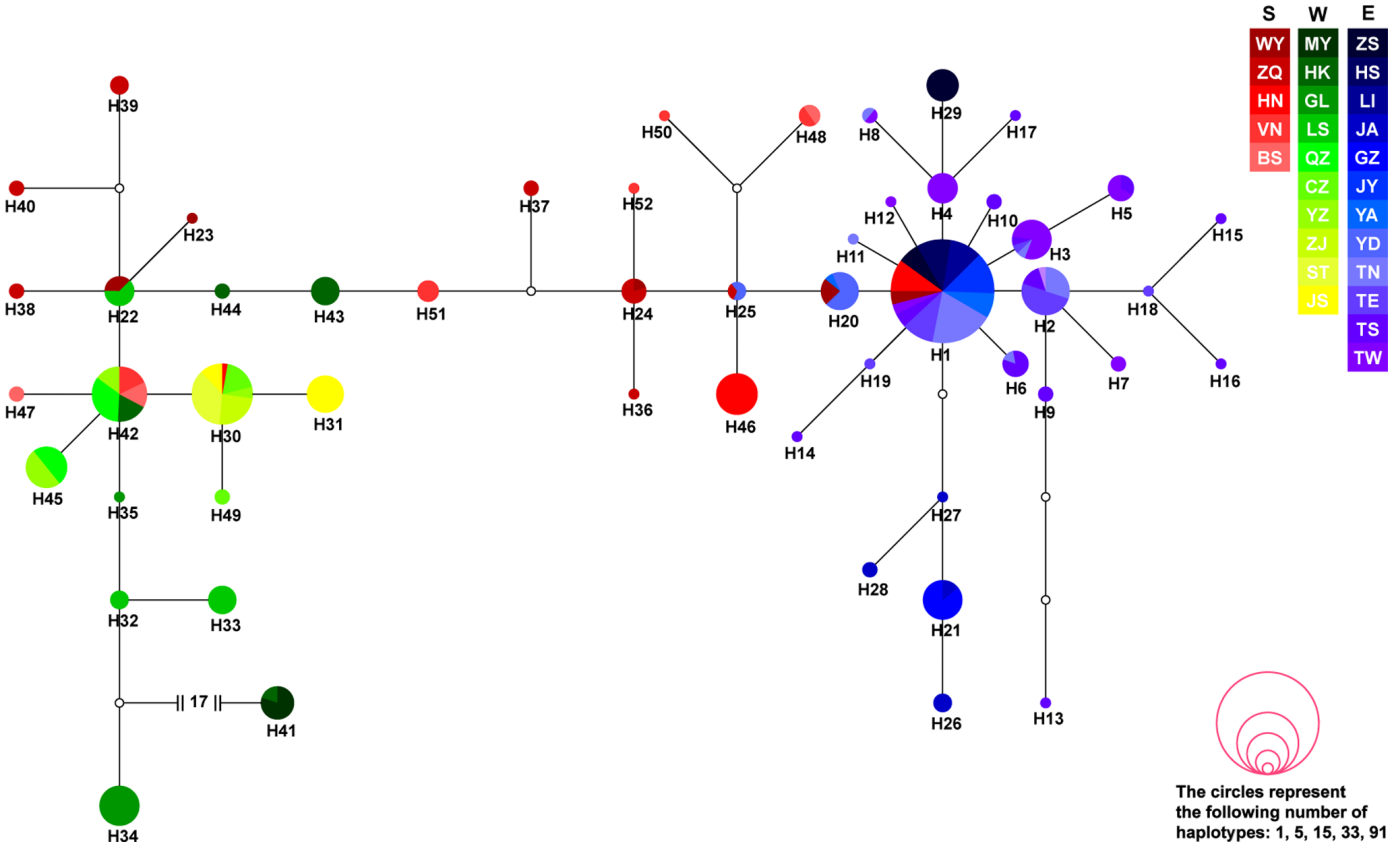
Network of 52 mitochondrial control region haplotypes from 390 individuals of *Naja atra*. The size of the circles is proportional to haplotype frequency; small open circles represent unsampled haplotypes. See Table 1 for sample site abbreviations.

**Figure 3:**
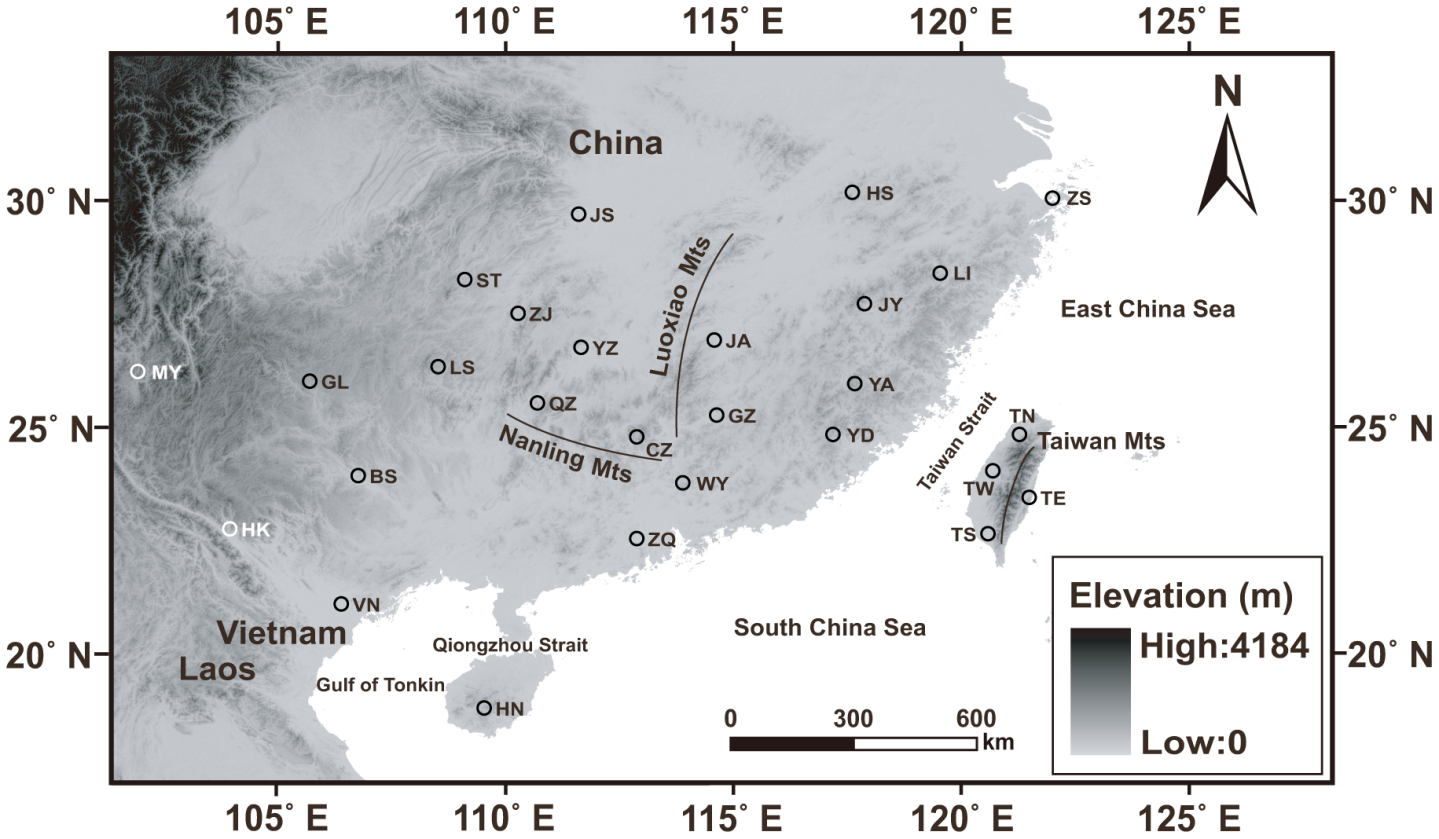
Sampling locations of *Naja atra* in this study. See Table 1 for sample site abbreviations. Solid lines indicate mountain series.

The phylogenetic tree resulting from ML revealed two clades (Fig. 1). This finding could be supported by the median-joining network (Fig. 2). Clade A included only one haplotype (H41) comprising 10 individuals from the two westernmost populations in the western region (Fig. 3), with eight of them from the Miyi population and other two from the Hekou population (Fig. 2). Clade B included haplotypes from sampling sites except Miyi. Clade A was divergent from Clade B by at least 17 mutation steps (Fig. 2).

Clade B had a complex pattern with two core haplotypes, H1 and H42, shared among geographically separated populations, and had broader mutation connections and higher frequencies (Fig. 2). H1 was shared by 11 populations, of which nine are in eastern China (including Taiwan) and two in Hainan and Wengyuan in southern China (Fig. 2). H42 was also shared by two southern populations in Vietnam and Baise and three western populations in Yongzhou, Quanzhou and Hekou (Fig. 2).

We defined three groups separated by the Luoxiao and Nanling Mountains: [eastern China], [southern China and Vietnam] and [western China] (Table 1). There was apparent geographical association among haplotypes within Clade B, with the haplotypes from the eastern group mainly on the right side of the network and the haplotypes from the western group mainly on the left side. The haplotypes from the southern group lay mainly in between (Fig. 2). All 19 haplotypes (H1–H19) except H1 found in the four Taiwan populations were not shared by any other population.

### Population structure and history

Using analysis of molecular variance, significant population structure was found for 27 populations and three groups (Φst = 0.76, *P*<0.0001). A significant proportion (34.1%) of the total variation in the mitochondrial DNA data could be attributable to differences among groups. A significant proportion of variation also occurred among populations within groups (41.9%) and within populations (24.0%).

Populations in eastern China show the genetic imprints of demographic expansion as inferred by a negative *Fs*, which was also supported by the mismatch distribution analysis and Rogers test of sudden population expansion (Table 1, [12]). The value of *τ* for the population group in eastern China was 1.824 based on a substitution rate of 0.0065 site^−1^ myr^−1^, and the expansion time was estimated to be 0.273 mya. The divergence time between the eastern population groups and the other two population groups was 0.363 mya according to BEAST (Fig. 1).

## Discussion

### Species origin, genetic structure and population differentiation

In the present study, cobras from the western (green), southern (red) and eastern (blue) population groups are at the base, in the middle and at the top of the ML phylogenetic tree, respectively (Fig. 1). This result, together with the fact that *N. atra* is the northeasternmost *Naja* species, supports the idea that cobras in the subgenus *Naja* spread from west to east [2]–[4], a pattern not following westwards, southwards or northwards adaptive radiations of species found in other parts of the world [13], [14]. Clade A (H41) was from two westernmost populations, one in Miyi and the other in Hekou, adjacent to the Tibetan Plateau. The haplotype was separated from the other 51 by at least 17 mutation steps (Fig. 2). This result is similar to an earlier study using the cyt *b* gene, where the haplotype (H31) from the Miyi and Hekou populations was even more distant from all other haplotypes, separated by at least 44 mutation steps [9].

An association between geographic region and haplotype within Clade B was apparent, with two “star” clusters represented by haplotypes mainly from either the eastern or the western populations (Fig. 2). Significant evolutionary clustering of haplotypes in different geographical regions has been frequently interpreted as evidence of past population fragmentation [15], [16]. The observed geographic pattern of haplotypes within Clade B could be largely attributed to dramatic climate changes during the late Pleistocene when cold and inclement conditions reoccurred. It is reasonable to assume that repeated range fragmentation and isolation, with subsequent founder effects and/or fixation events, followed by expansion of isolated populations (see below), probably reinforced evolution of *N. atra*, shaping the current genetic structure of populations.

### Barriers limiting gene flow

The haplotypes found in the four Taiwan populations were close to those found in the eastern mainland populations. All 19 haplotypes (H1–H19) found in Taiwan were clustered on the right side of the haplotype network. The 18 unique haplotypes were no more than four steps from the core haplotype H1 (Fig. 2), and nearly 1/3 (6/19) of the haplotypes in Taiwan were shared by the four populations on the island. This pattern indicates that the role of the mountains in central Taiwan in restricting gene flow is less important than suggested previously [11].

Similar to what has been observed between mainland and island populations in the northeastern (Zhoushan) and southern (Hainan) parts of the cobra's distributional range [9], [10], lineage sorting between mainland and Taiwan populations is incomplete (Fig. 1), because of the young age of the Taiwan Strait. The island of Taiwan was formed during the Miocene, and is separated from the southeast coast of mainland China by the Taiwan Strait, which is no more than 100 m deep and has been a land bridge during glacial periods. Climate change during the late Pleistocene (11,000 to 42,000 years ago) caused large-scale marine regression, resulting in a ∼130–180 meter fall of sea level. At that time, most parts of the Taiwan Strait changed into land, providing opportunity for terrestrial animals including *N. atra* to migrate between mainland China and Taiwan.

Populations on the two different sides of the Luoxiao and Nanling Mountains often do not share haplotypes, although they are geographically very close. This result is consistent with an earlier study using 12 microsatellite loci and the cyt *b* gene. The study shows that the two mountain ranges are important physical barriers limiting gene flow in *N. atra* [9]. The eastern and western populations of *N. atra* adjoin in Anhui, Hubei and Jiangxi provinces in the central-east part of China, indicating a “west–east division” in the species. Well differentiated evolutionary lineages and a similar “west–east division” have also been reported for other squamate reptiles such as the sharp-snouted pit viper *Deinagkistrodon acutus* [17], the short-tailed pit viper *Gloydius brevicaudus* [18] and the northern grass lizard *Takydromus septentrionalis* [19] based on mitochondrial DNA sequence data. It is worth noting that the presumed physical barriers such as the Luoxiao Mountains may not always act as barriers to migration. In *T. septentrionalis*, for example, all the haplotypes from the Yudu population east to the Luoxiao Mountains are clustered within the western lineage [19].

The phylogenetic tree showed a two-way dichotomy between Clades A and B, which provides no evidence that one clade is older than the other, as would be expected in a dispersal scenario. The two-way dichotomy might be the consequence of vicariant events subdividing widespread populations. More than a half of the total genetic variation occurred among the regions and among populations within a region, indicating high levels of geographical structuring and restricted gene flow. This population genetic structure could be largely due to the fact that this widespread snake has a limited ability to disperse. Herpetofauna including *N. atra* that are less likely to disperse are more prone to suffering substantial losses in genetic diversity resulting from habitat loss and fragmentation [20]–[23].

### Recent expansion of populations

The neutrality test and mismatch distribution analysis suggest that the eastern populations showed population expansion. This is also supported by the haplotype network (Fig. 2). Here, at least two conclusions can be drawn according to the “coalescent theory”: (1) the ancient haplotypes are in the center of the network, and (2) the ancient haplotypes have a wider distribution [24]. The eastern populations have a “star-like” topology with a core haplotype of H1, which are shared among 11 geographically separated populations, and have broader mutation connections and higher frequencies (Fig. 2). The characteristic “star-like” topology of haplotypes connecting with H1 indicates a recent demographic expansion [25], [26].

The expansion of eastern populations occurred some 0.273 million years ago. This is consistent with the result reported for *D. acutus*, a snake that has almost the same distributional range as *N. atra*, with populations of the former species in eastern China also experiencing expansion 0.226–0.252 million years ago [17]. Studies on these two species suggest that sympatric or co-distributed snakes may have similar population histories.

Prolonged exposure to temperatures lower than 9°C is lethal to *N. atra* [27]. Thus, it could be possible that the large-scale low temperature during Quaternary glaciation may have resulted in the disappearance of many ancient haplotypes. The subsequent last interglacial had a few relatively stable warm periods, though intensive climate changes also occurred. The population expansion of *N. atra* occurred in the early last interglacial, when was a global warming period, which provided the opportunity for cobras of this species to disperse, colonize, and undergo in situ genetic differentiation.

### Conservation implications

Studies on snake species with large and continuous ranges have generally shown the existence of independent management units for different lineages that should be taken into account for conservation plans [9]–[11], [17], [18], [28]–[30]. Given an association between geographic region and haplotype, the escapes or the uncontrolled releases of wild-caught individuals of *N. atra* will have the potential to disrupt lineage relationships. Therefore, we caution against the release of cobras into the wild unless their origins can be determined with confidence. The ex-situ conservation benefits gene flow and more easily reaches the minimum surviving quantity to maintain populations, but the genetic similarity between populations should be considered before executing such conservation plans. Besides the westernmost population group (Hekou and Miyi), three population groups ([eastern China], [southern China and Vietnam] and [western China]) separated by the Luoxiao and Nanling Mountains can be identified, but how much adaptive differentiation is present between these groups or within each group is currently unknown. Therefore, we also caution against long-distance transfers within any group, especially when environmental differences are apparent. Comparatively speaking, in-situ conservation by protecting and recovering habitats of *N. atra* is more sound and viable than other measures.

## Materials and Methods

### Sample collection and DNA sequencing

We collected 285 adult cobras larger than 900 mm snout-vent length from 23 localities covering the species' whole range in mainland China (including Zhoushan and Hainan Islands) and Vietnam (Fig. 3). Effort was made to avoid collecting more than one individual from the same site. The most distal 25 mm of the tail tip of each individual was excised using a sterilized scalpel. Tissue samples were preserved in 95% ethanol before they were deposited at Nanjing Normal University under voucher numbers identified by locality-haplotype numbers. Our experimental procedures complied with the current laws on animal welfare and research in China, and were approved by the Animal Research Ethics Committee of Nanjing Normal University (AREC 2004-04-020). The Forestry Departments of Anhui, Fujian, Guangdong, Guangxi, Guizhou, Hainan, Hunan, Jiangxi, Yunnan and Zhejiang provided permits for capturing cobras in China. The collection of Vietnamese specimens was conducted under the license from Vietnam National Museum of Nature, which was accepted by the Animal Research Ethics Committee of Nanjing Normal University. We sequenced the mitochondrial control region from the 285 individuals, and obtained an additional 18 sequences unique to Taiwan from GenBank [11].

Preserved tissue samples were used to extract total genomic DNA using EasyPure Genomic DNA Extraction Kit (TransGen Biotech). DNA was resuspended in TE buffer (10 mM Tris-HCl, pH 8.0, 0.1 mM EDTA) and stored at −80°C until ready for use. We used primers PL1 (5′- CCA CAA AGC ATT GTT CTT GTA AAC C T-3′) and PH1 (5′-GAA GCT TGC ATG TAT AAG TAG GG-3′) [11] to amplify the control region. Mitochondrial DNA was amplified from template DNA in 50 µl reactions using a hot-start method in a thermal cycler with a 3-min denaturing step at 94°C followed by 40 cycles of denaturing for 30 sec at 94°C, primer annealing for 40 sec at 55°C and elongation for 70 sec at 72°C with a final 10-min elongation step at 72°C. Cycle sequencing was conducted with primer PL1 and PH1.

The mtDNA of cobras has two control regions (CR1 and CR2). CR1 is located between tRNA-Pro and tRNA-Phe, containing the replication origin of heavy chain and the transcription start site of duplexes; CR2 is located between tRNA-Ile and tRNA-Leu [31]. In this study, we used primers PL1 and PH1 to sequence the CR1 region, which was also used for Taiwan-originated *N. atra* [11]. This pair of primers was designed according to the conserved regions of tRNA-Pro and tRNA-Phe at the end of CR1.

### Data analysis

We compiled and aligned sequences using MEGA 5.1 [32]. We used DNASP 5.0 to identify haplotypes [33] and estimate genetic diversity within populations by haplotype (*h*) and nucleotide diversities (*π*) [34]. We reconstructed a phylogenetic tree based on the maximum likelihood (ML) method, using the king cobra *Ophiophagus hannah* and the banded krait *Bungarus fasciatus* (GenBank Accession number EU921899 and EU579523 respectively) as outgroups. ML analysis was carried out by a heuristic search of 10 random addition analyses with tree-bisection-reconnection (TBR) branch swapping using PAUP^*^ 4.0b10 [35]. The TIM + I + G substitution model [36] was selected by ModelTest 3.8 [37] based on the Akaike information criterion (AIC) [38]. The confidence level of the nodes in the ML tree was estimated using 1000 bootstrap pseudoreplicates. We also conducted a median-joining network (MJN) approach [39] to depict relationships among the haplotypes. This approach has been shown to yield the best-resolved genealogies relative to other rooting and network procedures [40]. The MJN was estimated using NETWORK 4.5.0.0 [39].

We performed hierarchical analysis of molecular variance (AMOVA) in ARLEQUIN 3.5 [41] with 10000 permutations to examine partitioning of genetic diversity within and among populations. We used mismatch distributions to test demographic signatures of population expansions within mtDNA lineages [12]. To compare observed distributions with those expected under the expansion model, we calculated the sum of squared deviation (*SSD*) and the Harpending's raggedness index [42]. Tajima's *D* test [43] and Fu's *Fs* test [44] were used to test equilibrium of the populations in ARLEQUIN 3.5. The statistics were expected to have significantly large negative values under demographic expansion. The equation *τ* = 2*ut* [45] was used to estimate the approximate expansion time in generations (*t*), where *τ* is the date of the growth or decline measured in units of mutational time and *u* is the mutation rate per sequence and per generation. The approximate time of expansion in years was calculated by multiplying *t* by the generation time of *N. atra*, estimated as four years based on the approximate time at which animals become mature [16], [46]. The substitution rate was estimated as 0.65%/myr as adopted in a similar study on *D. acutus* [17]. We used a Bayesian approach to estimate divergence time with BEAST 1.8.0 [47]. We used BEAUti to set criteria for the analysis. We used the Bayes factor calculated by Tracer 1.5 to check for convergence of Markov chain Monte Carlo (MCMC) and adequate effective sample sizes (> 200) after the first 20% of generations had been discarded as burn-in. The MCMC simulation was run for 10,000,000 generations, and trees were sampled every 1000 generations. We used a Yule tree prior with an uncorrelated lognormal relaxed clock. The final joint sample was used to estimate the maximum clade credibility tree in TreeAnnotator 1.8.0 with a burn-in of 1000 trees. We evaluated and edited final trees in FigTree 1.3.1.

## References

[1] Wallach V, Wüster W, Broadley DG (2009) In praise of subgenera: taxonomic status of cobras of the genus *Naja* Laurenti (Serpentes: Elapidae). Zootaxa 2236: 26–36.

[2] Ineich I (1995) Etat actuel de nos connaissances sur la classification des serpents venimeux. Bull Soc Herpétol France 75/76: 7–24.

[3] Minton SA (1986) Origins of poisonous snakes: evidence from plasma and venom proteins. In: Harris JB, editor. Natural toxins: animal, plant and microbial. Oxford: Clarendon Press. pp. 3–21.

[4] Wüster W, Crookes S, Ineich I, Mane Y, Pook CE, et al (2007) The phylogeny of cobras inferred from mitochondrial DNA sequences: evolution of venom spitting and the phylogeography of the African spitting cobras (Serpentes: Elapidae: *Naja nigricollis* complex). Mol Phylogenet Evol 45: 437–453. 17870616 10.1016/j.ympev.2007.07.021

[5] Zhao EM (1998) *Naja atra*. In: Zhao EM, editor. China red data book of endangered animals, Amphibia and Reptilia. Beijing: Science Press. pp. 274–276.

[6] Slowinski JB, Wüster W (2000) A new cobra (Elapidae: *Naja*) from Myanmar (Burma). Herpetologica 56: 257–270.

[7] Wüster W (1996) The status of the cobras of the genus *Naja* Laurenti, 1768 (Reptilia: Serpentes: Elapidae) on the island of Sulawesi. Snake 27: 85–90.

[8] Wüster W, Thorpe RS (1992) Asiatic cobras: population systematics of the *Naja naja* species complex (Serpentes: Elapidae) in India and Central Asia. Herpetologica 48: 69–85.

[9] Lin LH, Qu YF, Li H, Zhou KY, Ji X (2012) Genetic structure and demographic history should inform conservation: Chinese cobras currently treated as homogenous show population divergence. PLoS One 7: e36334. 22558439 10.1371/journal.pone.0036334 PMC3338645

[10] Lin LH, Zhao Q, Ji X (2008) Conservation genetics of the Chinese cobra (*Naja atra*) investigated with mitochondrial DNA sequences. Zool Sci 25: 888–893. 19267597 10.2108/zsj.25.888

[11] Lin HC, Li SH, Fong J, Lin SM (2008) Ventral coloration differentiation and mitochondrial sequences of the Chinese Cobra (*Naja atra*) in Taiwan. Conserv Genet 9: 1089–1097.

[12] Rogers AR (1995) Genetic evidence for a Pleistocene population explosion. Evolution 49: 608–615. 28565146 10.1111/j.1558-5646.1995.tb02297.x

[13] Forcina G, Panayides P, Guerrini M, Nardi F, Gupta BK, et al (2012) Molecular evolution of the Asian francolins (*Francolinus*, Galliformes): a modern reappraisal of a classic study in speciation. Mol Phylogenet Evol 65: 523–534. 22828178 10.1016/j.ympev.2012.07.006

[14] Zink RM, Groth JG, Vázquez-Miranda H, Barrowclough GF (2013) Phylogeography of the California Gnatcatcher (*Polioptila californica*) using multilocus DNA sequences and ecological niche modeling: implications for conservation. Auk 130: 449–458.

[15] Avise JC (1987) Intraspecific phylogeography: the mitochondrial DNA bridge between population genetics and systematics. Annu Rev Ecol Evol Syst 18: 489–522.

[16] Templeton AR (1998) Nested clade analyses of phylogeographic data: testing hypotheses about gene flow population history. Mol Ecol 7: 381–397. 9627999 10.1046/j.1365-294x.1998.00308.x

[17] Huang S, He SP, Peng ZG, Zhao K, Zhao EM (2007) Molecular phylogeography of endangered sharp-snouted pitviper (*Deinagkistrodon acutus*; Reptilia, Viperidae) in Mainland China. Mol Phylogenet Evol 44: 942–952. 17643319 10.1016/j.ympev.2007.05.019

[18] Ding L, Gan XN, He SP, Zhao EM (2011) A phylogeographic, demographic and historical analysis of the short-tailed pit viper (*Gloydius brevicaudus*): evidence for early divergence and late expansion during the Pleistocene. Mol Ecol 20: 1905–1922. 21438932 10.1111/j.1365-294X.2011.05060.x

[19] Cai Y, Yan J, Xu XF, Lin ZH, Ji X (2012) Mitochondrial DNA phylogeography reveals a west-east division of the northern grass lizard (*Takydromus septentrionalis*) endemic to China. J Zool Syst Evol Rese 50(2): 137–144.

[20] Bouzat JL, Cheng HH, Lewin HA, Westemeier RL, Brawn JD, et al (1998) Genetic evaluation of a demographic bottleneck in the greater prairie chicken. Conserv Biol 12: 836–843.

[21] Lin CX, Du Y, Qiu QB, Ji X (2007) Relatively high but narrow incubation temperatures in lizards depositing eggs in warm and thermally stable nests. Acta Zool Sin 53: 437–445.

[22] Taylor AC, Sherwin WB, Wayne RK (1994) Genetic variation of microsatellite loci in a bottlenecked species: the northern hairynosed wombat *Lasiorhinus krefftii* . Mol Ecol 3: 277–290. 7921355 10.1111/j.1365-294x.1994.tb00068.x

[23] Wisely SM, Buskirk SW, Fleming MA, McDonald DB, Ostrander EA (2002) Genetic diversity and fitness in black-footed ferrets before and during a bottleneck. J Hered 93: 231–237. 12407208 10.1093/jhered/93.4.231

[24] Posada D, Crandall KA (2001) Intraspecific gene genealogies: trees grafting into networks. Trends Ecol Evol 16: 37–45. 11146143 10.1016/s0169-5347(00)02026-7

[25] Avise JC (2000) Phylogeography: the history and formation of species. Cambridge: Harvard University Press.

[26] Slatkin M, Hudson RR (1991) Pairwise comparisons of mitochondrial sequences in stable and exponentially growing populations. Genetics 129: 555–562. 1743491 10.1093/genetics/129.2.555 PMC1204643

[27] Ji X, Chen HL, Du WG, Zhu BQ (2002) Radiotelemetry of thermoregulation and thermal tolerance of Chinese cobras (*Naja atra*) overwintering in a laboratory enclosure. Acta Zool Sin 48: 591–598.

[28] Chiucchi JE, Gibbs HL (2010) Similarity of contemporary and historical gene flow among highly fragmented populations of an endangered rattlesnake. Mol Ecol 19: 5345–5358. 20964755 10.1111/j.1365-294X.2010.04860.x

[29] Huang S, Liu SY, Guo P, Zhang YP, Zhao EM (2009) What are the closest relatives of the hot-spring snakes (Colubridae, *Thermophis*), the relict species endemic to the Tibetan Plateau? Mol Phylogenet Evol 51: 438–446. 19249375 10.1016/j.ympev.2009.02.013

[30] Sallaberry-Pincheira N, Garin CF, González-Acuña D, Sallaberry MA, Vianna JA (2011) Genetic divergence of Chilean long-tailed snake (*Philodryas chamissonis*) across latitudes: conservation threats for different lineages. Divers Distrib 17: 152–162.

[31] Yan J, Li HD, Zhou KY (2008) Evolution of the mitochondrial genome in snakes: Gene rearrangements and phylogenetic relationships. BMC Genomics 9: 569. 19038056 10.1186/1471-2164-9-569 PMC2632649

[32] Kumar S, Tamura K, Nei M (2004) MEGA3: integrated software for molecular evolutionary genetics analysis and sequence alignment. Brief Bioinform 5: 50–163. 10.1093/bib/5.2.150 15260895

[33] Librado P, Rozas J (2009) DnaSP v5: A software for comprehensive analysis of DNA polymorphism data. Bioinformatics 25: 1451–1452. 19346325 10.1093/bioinformatics/btp187

[34] Nei M (1987) Molecular evolutionary genetics. New York: Columbia University Press.

[35] Swofford DL (2003) PAUP* phylogenetic analysis using parsimony (*and other methods), Version 4. Massachusetts: Sinauer Associates.

[36] Tamura K, Nei M (1993) Estimation of the number of nucleotide substitutions in the control region of mitochondrial DNA in humans and chimpanzees. Mol Biol Evol 10: 512–526. 8336541 10.1093/oxfordjournals.molbev.a040023

[37] Posada D, Crandall KA (1998) Modeltest: testing the model of DNA substitution. Bioinformatics 14: 817–818. 9918953 10.1093/bioinformatics/14.9.817

[38] Akaike H (1974) A new look at the statistical model identification. IEEE T Automat Contr 19: 716–723.

[39] Bandelt HJ, Forster P, Rohl A (1999) Median-joining networks for inferring intraspecific phylogenies. Mol Biol Evol 16: 37–48. 10331250 10.1093/oxfordjournals.molbev.a026036

[40] Cassens I, Waerebeek K, Best PB, Crespo EO, Reyes J, et al (2003) The phylogeography of dusky dolphins (*Lagenorhynchus obscurus*): a critical examination of network methods and rooting procedure. Mol Ecol 12: 1781–1792. 12803631 10.1046/j.1365-294x.2003.01876.x

[41] Excoffier L, Laval G, Schneider S (2005) ARLEQUIN (version 3.0): an integrated software package for population genetics data analysis. Evol Bioinform Online 1: 47–50. PMC2658868 19325852

[42] Harpending HC (1994) Signature of ancient population growth in a low-resolution mitochondrial DNA mismatch distribution. Hum Biol 66: 591–600. 8088750

[43] Tajima F (1989) Statistical method for testing the neutral mutation hypothesis by DNA polymorphism. Genetics 123: 585–595. 2513255 10.1093/genetics/123.3.585 PMC1203831

[44] Fu YX (1997) Statistical tests of neutrality of mutations against population growth, hitchhiking and background selection. Genetics 147: 915–925. 9335623 10.1093/genetics/147.2.915 PMC1208208

[45] Rogers AR, Harpending H (1992) Population growth makes waves in the distribution of pairwise genetic differences. Mol Biol Evol 9: 552–569. 1316531 10.1093/oxfordjournals.molbev.a040727

[46] Huang S, Huang JT (2003) Artificial propagation of the five-paced pitviper (*Deinagkistrodon acutus*). Acta Zool Sin 49: 854–857.

[47] Drummond AJ, Rambaut A (2007) BEAST: Bayesian evolutionary analysis by sampling trees. BMC Evol Biol 7: 214. 17996036 10.1186/1471-2148-7-214 PMC2247476

